# Type less, think more

**DOI:** 10.1016/j.patter.2024.101076

**Published:** 2024-10-11

**Authors:** Andrew L. Hufton, Alejandra Alvarado

**Affiliations:** Editor-in-Chief, *Patterns*; Scientific Editor, *Patterns*

## Main text

In this issue of *Patterns*, we explore the impact that large language models (LLM), such as ChatGPT, are having on scientific writing and skill development. Now, almost 2 years after the original release of ChatGPT, we reached out to a set of *Patterns* authors and others to see how these models are changing teaching methods and influencing writing and learning skills.

For this issue’s People of Data article, we asked three scientists who recently published at *Patterns* to share their experiences using AI tools to prepare scientific manuscripts. Overall, they were positive about the impact AI tools have had on their writing process. They noted that ChatGPT saved time with routine writing tasks such as tweaking text to improve readability or correcting grammar. This is consistent with other views (Pividori and Greene, 2024, *J Am Med Inform Assoc*; Noorden and Perkel 2023, *Nature*), but while the efficiency brought to the tedious process of scientific writing by AI is undeniable, the buzz comes with some caution. All three authors indicated that they were careful to use AI only to proofread existing text and for routine formatting tasks, not for generating new content. Yinqi Bai worried that “the misuse of AI tools—such as asking them to generate research conclusions or to cite fabricated references—could significantly mislead both reviewers and readers.”

It is worth noting that we were able to invite these views because each of these authors acknowledged using AI during manuscript writing. This is required by Elsevier's AI use policies, but we nonetheless thank these authors for their openness. It is no surprise to us that ethical, thoughtful use goes hand-in-hand with transparency. As the saying goes, sunlight is the best disinfectant. Being transparent with your co-authors and the journals to which you submit about how you use AI tools is the best way to ensure responsible use.

Looking more from the perspective of education, Brent Anders argues that students need to learn to generate AI text that is actually usable and directly encourages educators to help students develop AI knowledge. He argues that skills will not be lost when using AI to write. Instead, skills will shift, from learning to write “in the traditional way,” to learning how to effectively edit AI-generated text and write effective prompts that generate good text in the first place. Anders makes a compelling case that future job seekers will need to acquire AI skills for professional advancement.

J. Scott Christianson, in a parallel Opinion, argues that trying to detect AI-generated content in classroom assignments is futile anyway. AI-produced content will simply adapt, ultimately creating a no-win situation, especially for teachers and educators. Instead of policing AI text, Christianson proposes a transformation in the way we teach, consciously rethinking writing assignments and incorporating AI.

At *Patterns*, we seek to feature many exciting new developments in the use of language models, but we would also like to remind our readers and authors to use AI as an assistant and not as a substitution for human writing. Be thoughtful, be cautious, and be transparent. Keep in mind as well that AI use is strictly not allowed during peer review (See our September editorial). Core scientific contributions must remain human, and by leveraging AI tools responsibly, we hope researchers can reach a point where writing a scientific manuscript requires less typing and more thinking.

